# X-ray evaluation of failed unicompartmental knee replacement

**DOI:** 10.1308/003588412X13373405387096

**Published:** 2012-05

**Authors:** YD Kamat, NW Bradley

**Affiliations:** Royal Surrey County Hospital NHS Foundation Trust,UK

## BACKGROUND

The most common causes of long-term failure of the Oxford medial unicompartmental knee arthoplasty (UKR) are the development of lateral compartment osteoarthritis, and component loosening and infection.^1,2^ Stress radiographs can be useful in assessing the lateral compartment either before considering unicompartmental replacement or in the investigation of a painful UKR.

## TECHNIQUE

**Figure 1:**
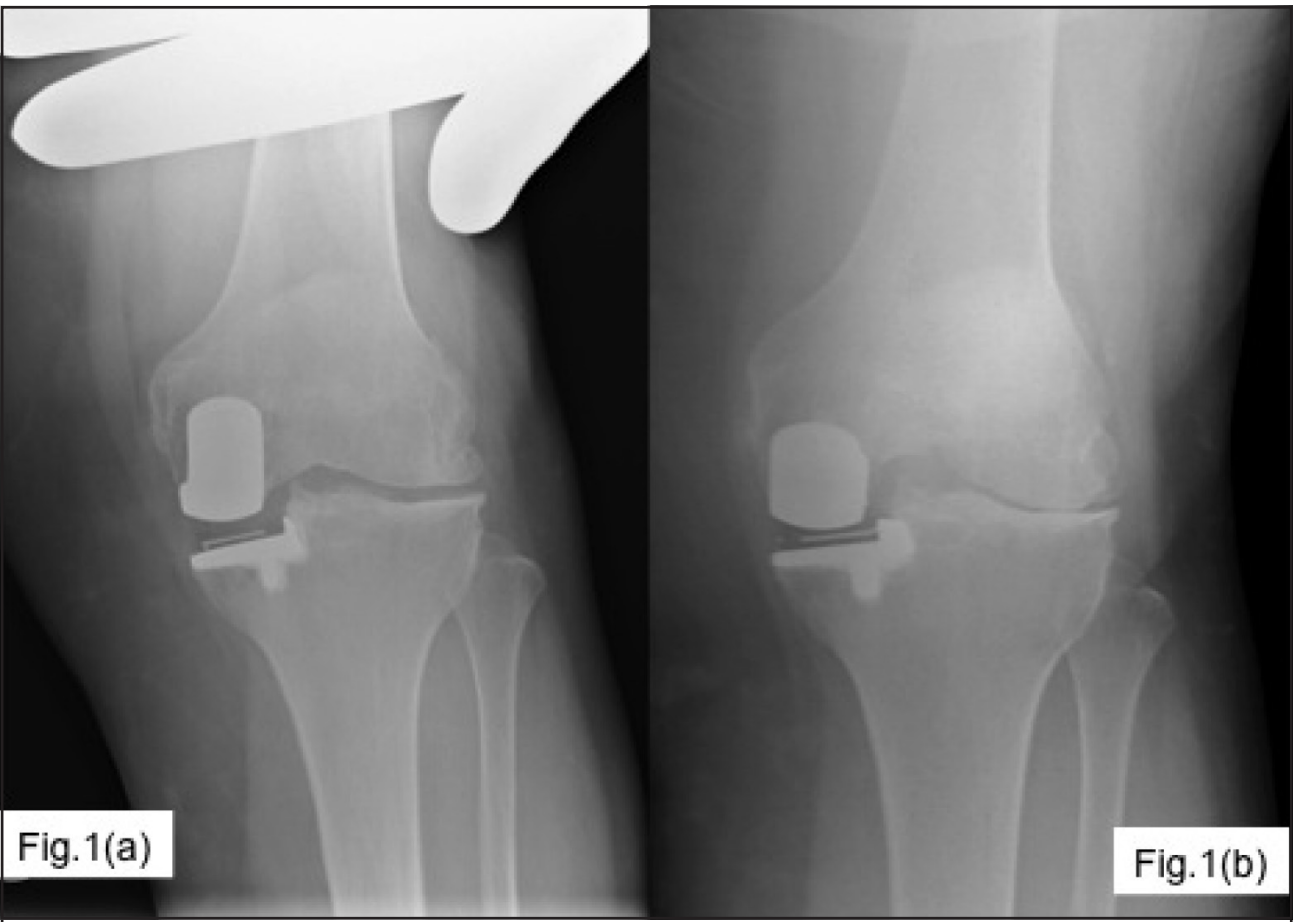
X-ray evaluation of a painful unicompartmental knee replacement. 1(a): AP valgus stress X-ray, 1(b): Lyon Schuss view.

The Lyon Schuss view (an anteroposterior weight-bearing radiograph taken with the knee in 20–30 degrees flexion) offers greater accuracy in determining moderate to severe loss of joint space compared with the standard weight-bearing view with the knee fully extended^3,4^. This is particularly so in lateral compartment arthritis where the pattern of disease is different, ie the posterior part of the compartment is affected. Corresponding appearances of the same joint with a AP valgus stress view (Fig 1a) and Lyon Schuss view (Fig 1b) are seen in the adjoining figure. Use of the Lyon Schuss radiographic views when evaluating causes of failed UKR has not previously been described.

## DISCUSSION

We recommend the Lyon Schuss view as a primary investigation for painful UKR as it instantly establishes the most common cause of failure instantly. The Lyon Schuss view also eliminates the risk of radiation exposure to staff and inconsistency of the applied force associated with stress views.^5^
